# miR-545 promotes colorectal cancer by inhibiting transferring in the non-normal ferroptosis signaling

**DOI:** 10.18632/aging.203801

**Published:** 2021-12-26

**Authors:** Sixin Zheng, Lingling Hu, Qingwen Song, Yuqiang Shan, Guang Yin, Hanzhang Zhu, Wencheng Kong, Chunhua Zhou

**Affiliations:** 1 Department of General Surgery, Affiliated Hangzhou First People Hospital, Zhejiang University School of Medicine, Hangzhou City, Zhejiang Province 310006, PR China; 2 Department of Medical Quality Management, Affiliated Hangzhou First People Hospital, Zhejing University School of Medicine, Hangzhou City, Zhejiang Province 310006, PR China

**Keywords:** miR-545, colorectal cancer, transferrin, ferroptosis

## Abstract

In this study, we examined whether and how miR-545 modulates ferroptosis in colorectal cancer (CRC). HT-29 and HCT-116 human CRC cell viability was examined using a CCK-8 assay and malondialdehyde (MDA) and Fe^2+^ levels were measured after treatment with the ferroptosis inducers Eradicator of Ras and ST (erastin) and Ras selective lethal 3 (RSL3) with or without miR-545 overexpression or knockdown vectors. Our results demonstrate that miR-545 overexpression inhibited, while miR-545 knockdown further increased, erastin and RSL3-induced upregulation of MDA, reactive oxygen species (ROS), and Fe^2+^ levels. Similarly, miR-545 overexpression partially reversed, while miR-545 knockdown enhanced, the erastin and RSL3-induced reduction in HT-29 and HCT-116 cell survival rates. Transferrin (TF) was identified as a target gene of miR-545. To determine whether miR-545 suppresses ferroptosis via TF, we overexpressed TF in HT-29 and HCT-116 cells. We found that TF overexpression blocked miR-545-induced changes in ROS, MDA, and Fe^2+^ levels in HT-29 and HCT-116 cells, thereby inducing CRC cell death. An *in vivo* assay showed that inhibition of miR-545 decreased tumor growth in nude mice treated with erastin. Together, these findings indicate that miR-545 promotes CRC cell survival by suppressing TF.

## INTRODUCTION

Colorectal cancer (CRC) is one of the most common digestive tract cancers, and its incidence rate is increasing year by year [1]. Multiple genetic and environmental factors modulate the occurrence and development of CRC [2, 3]. However, the pathogenesis of CRC remains largely unclear, and additional studies are needed to identify key molecules involved in the growth and metastasis of CRC.

Ferroptosis is a programmed cell death which depends on iron and lipid peroxidation [4]. It affects various morphological, biochemical, and genetic processes in a distinct manner compared to apoptosis, autophagy, and necrosis [5, 6]. Eradicator of Ras as well as ST (erastin) and Ras selective lethal 3 (RSL3) are two important ferroptosis-inducing compounds [7, 8]. In the canonical pathway, glutathione peroxidases (GPXs) induce ferroptosis by inactivating the major membrane mechanisms that protect against peroxidation damage [9]. Glutathione (GSH) and antioxidant enzymes can decrease the levels of reactive oxygen species (ROS) and then reduce the lipid peroxidation products, such as malondialdehyde (MDA), 4-hydroxynonenal (4-HNE) [10]. In addition, non-canonical ferroptosis is initiated when the labile iron pool (LIP) increases [9]. Specifically, abnormal activation of heme oxygenase 1 (HMOX1), reduced the expression of ferroportin, and enhanced the expression of transferrin expression [9].

MicroRNAs (miRNAs, miRs) are a class of small, non-coding RNAs which suppress the translation of target messenger RNAs [11, 12]. Accumulating evidence has shown that miRs play key roles in tumor initiation, progression, and metastasis [13, 14]. Recently, miR-137 was found to participate in ferroptosis by suppressing glutaminolysis, suggesting that it might have therapeutic potential in melanoma [15]. In CRC, miR-424-5p acts as a tumor suppressor by inhibiting ACSL4 [16]. A previous study demonstrated that LINC00342 affects various cellular processes in colon adenocarcinoma (COAD) both *in vivo* and *in vitro* by modulating miR-545-5p/MDM2 expression [17]. However, whether miR-454 is involved in ferroptosis-related cell death in CRC remains unknown.

## RESULTS

### miR-545 was elevated in CRC cells

**Figure 1:**
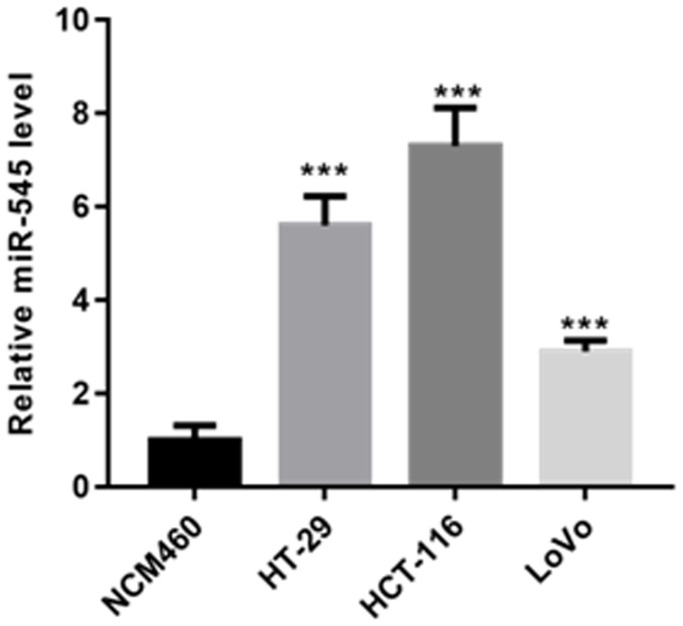
RT-PCR analysis demonstrated that the level of miR-545 was significantly increased in HT-29, HCT-116 and LoVo cells than that of NCM460 cells.

First, we evaluated the expression of miR-545 in CRC cells. In contrast to that of NCM460 cells, miR-545 was significantly increased in HT-29, HCT-116 and LoVo cells (Figure 1).

### miR-545 enhances erastin and RSL3-induced ferroptosis in CRC cells

**Figure 2:**
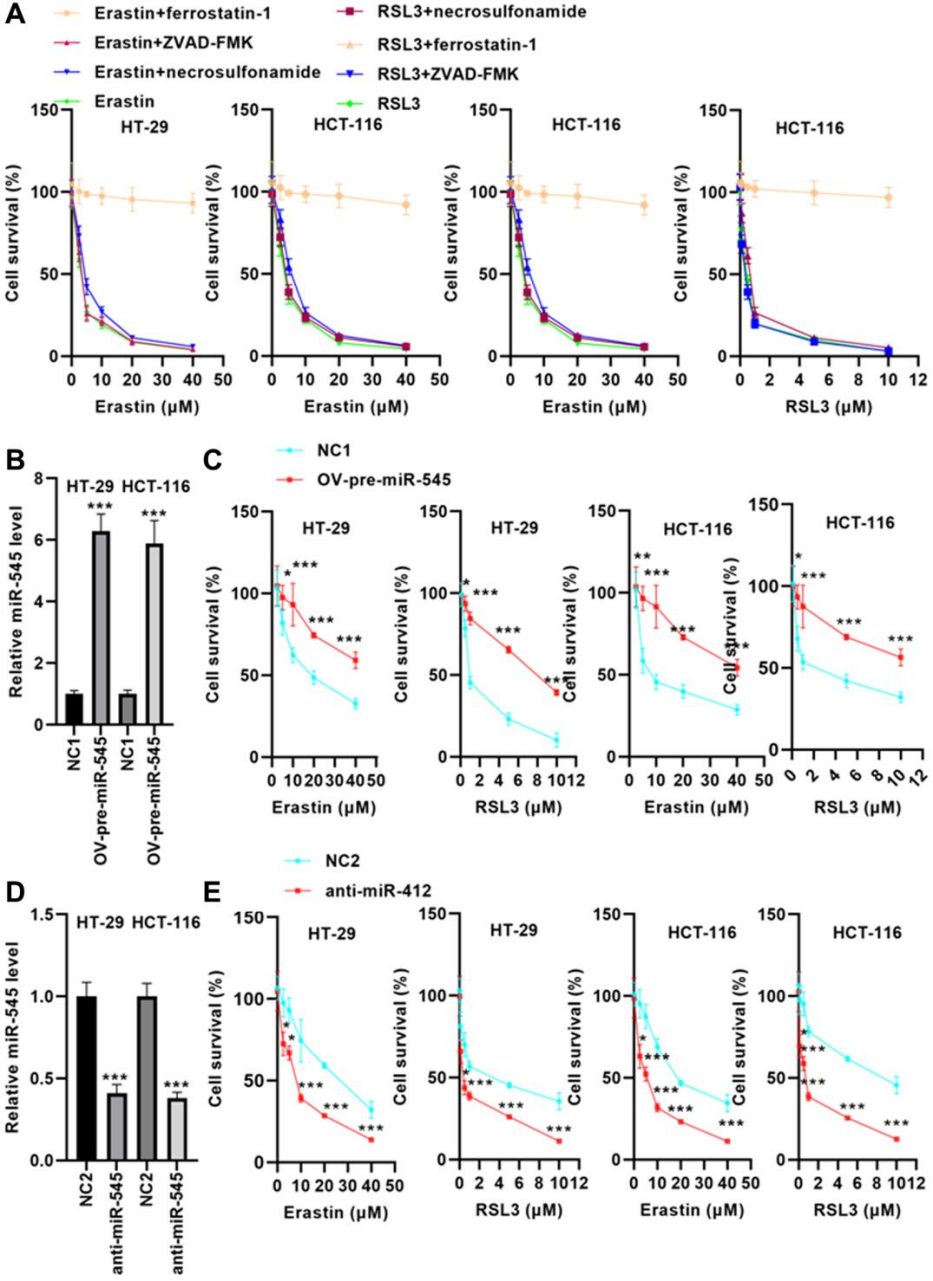
**miR-545 increased erastin and RSL3-induced ferroptosis in HT-29 and HCT-116 cells.** (**A**) Erastin and RSL3 significantly decreased cell survival rate in a dose-dependent manner. (**B**) RT-PCR showed that transfection with OV-pre-miR-545 significantly increased miR-545 levels in HT-29 and HCT-116 cells. (**C**) Pretreatment with miR-545 significantly increased HT-29 and HCT-116 cell survival rates. (**D**) Transfection with anti-miR-545 significantly reduced miR-545 levels in HT-29 and HCT-116 cells. (**E**) CCK-8 assay demonstrated that miR-545 knockdown further reduced the erastin and RSL3-induced decrease in cell survival rate. ^*^p < 0.05, ^**^p < 0.01, ^***^p < 0.001 vs. as indicated.

Erastin and RSL3 were used to treat HT-29 and HCT-116 cells. The results showed that erastin and RSL3 significantly reduced HT-29 and HCT-116 cell survival by the dose dependence way (Figure 2A). Furthermore, suppression of ferroptosis via ferrostatin-1 abolished CRC cell death induced by erastin and RSL3, but preincubation with apoptosis inhibitor, ZVAD-FMK and necroptosis inhibitor, necrosulfonamide did not (Figure 2A). These data indicate that CRC cells were susceptible to ferroptosis inducers.

To evaluate whether miR-545 regulates ferroptosis, we transfected CRC cells with an OV-pre-miR-545 plasmid. RT-PCR analysis indicated that OV-pre-miR-545 significantly upregulated miR-545 levels in CRC cells (Figure 2B). After incubation with erastin and RSL3, cell survival rate was analyzed. As shown in Figure 2C, pretreatment with miR-545 significantly increased CRC cell survival rates.

In contrast, transfection with anti-miR-545 significantly reduced miR-545 levels in CRC cells (Figure 2D). The CCK-8 assay demonstrated that suppression of miR-545 reduced the survival rate of CRC cells after erastin and RSL3 treatment (Figure 2E). These data suggest that miR-545 may increase CRC cell survival by inhibiting ferroptosis.

### miR-545 decreases lipid oxidation and iron accumulation in CRC cells

**Figure 3:**
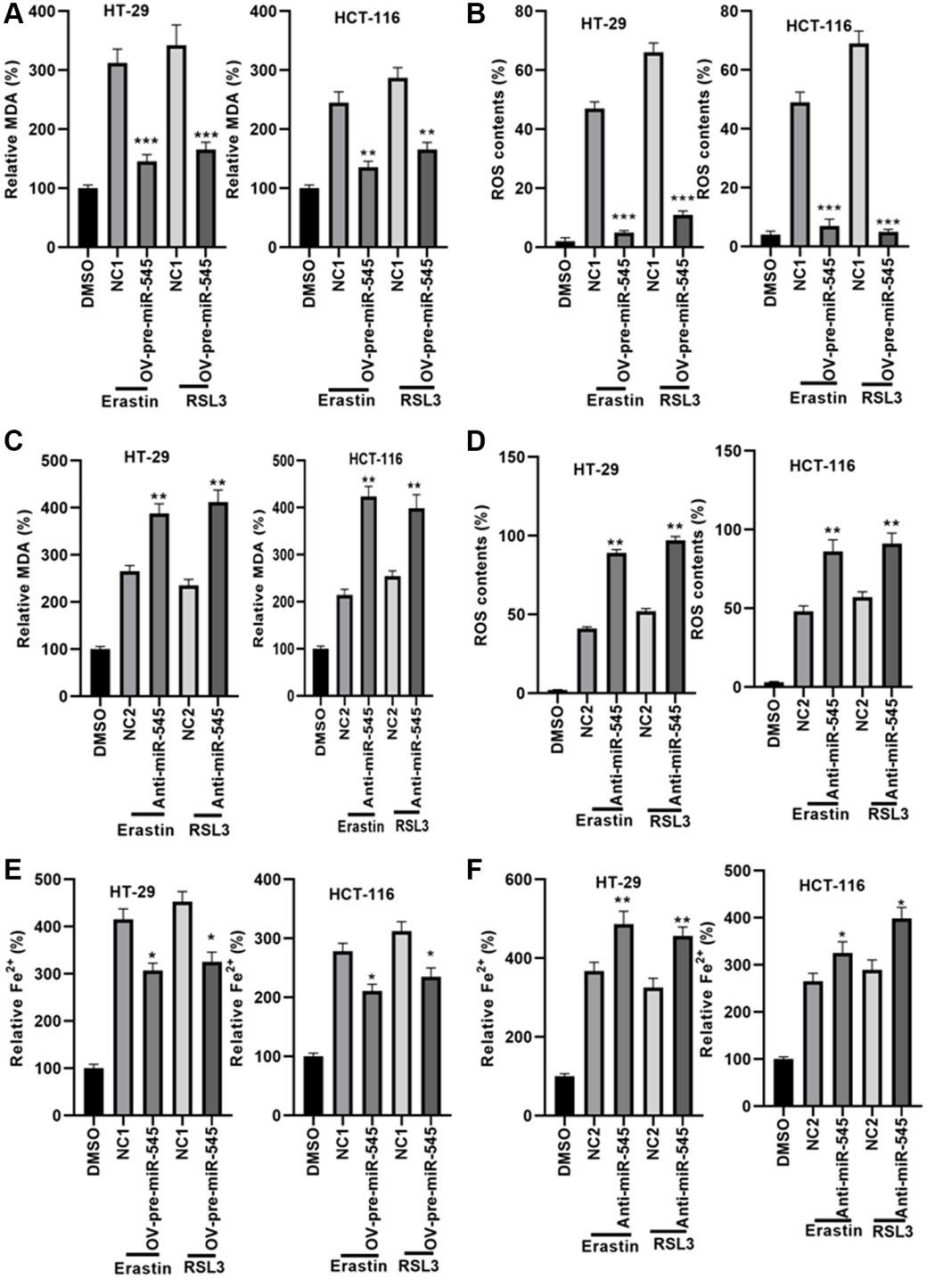
**miR-545 decreased lipid oxidation and iron accumulation in HT-29 and HCT-116 cells.** An erastin and RSL3-induced increase in MDA and ROS levels was significantly reduced in HT-29 and HCT-116 cells transfected with OV-pre-miR-545 (**A**), while inhibition of miR-545 further increased MDA and ROS levels (**B**), in both HT-29 and HCT-116 cells. ROS levels were reduced in HT-29 and HCT-116 cells transfected with OV-pre-miR-545 (**C**), but inhibition of miR-545 further increased ROS levels (**D**), in both HT-29 and HCT-116 cells. (**E**) Levels of ferrous iron (Fe2+) were decreased in HT-29 and HCT-116 cells transfected with OV-pre-miR-545 following treatment with erastin and RSL3. (**F**) Anti-miR-545 further increased Fe2+ levels in erastin and RSL3-treated HT-29 and HCT-116 cells. ^*^p < 0.05, ^**^p < 0.01, ^***^p < 0.001 vs. as indicated.

Next, we explored the effects of miR-545 on lipid oxidation and iron accumulation, two key ferroptosis mechanisms, in CRC cells. Our data showed that transfection with OV-pre-miR-545 inhibited, while anti-miR-545 increased, erastin and RSL3-induced increases in MDA levels in CRC cells (Figure 3A and 3B). Similarly, erastin and RSL3-induced increases in ROS levels decreased when miR-545 was overexpressed and further increased when miR-545 was inhibited in CRC cells (Figure 3C and 3D). Furthermore, ferrous iron (Fe^2+^) levels decreased in CRC cells transfected with OV-pre-miR-545 after erastin and RSL3 treatment (Figure 3E). In contrast, anti-miR-545 further increased Fe^2+^ levels in erastin and RSL3-treated CRC cells (Figure 3F). The above findings confirmed that miR-545 inhibited ferroptosis by regulating lipid oxidation and iron accumulation.

### miR-545 targets the transferrin gene

**Figure 4:**
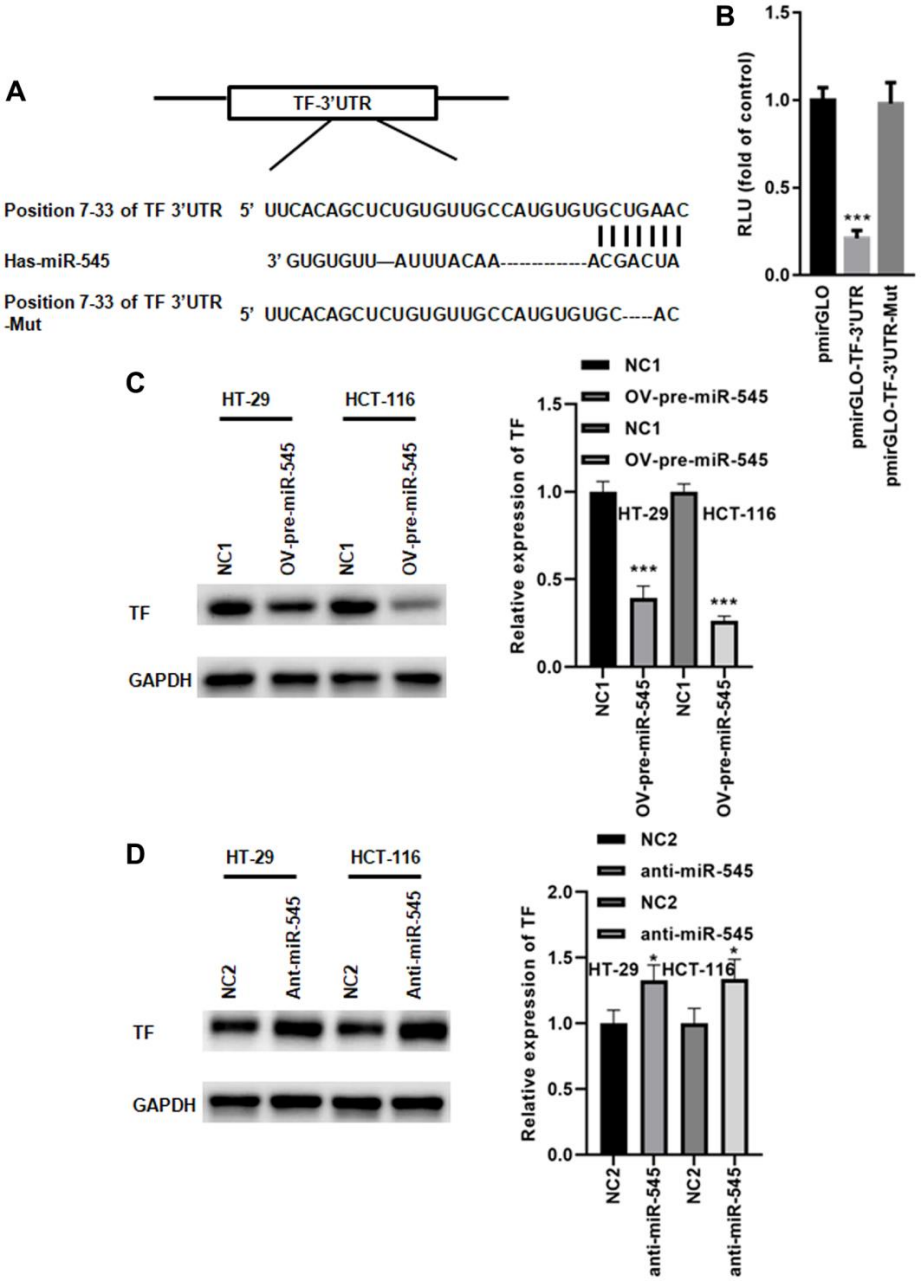
**miR-545 targets the transferrin gene.** (**A**) A conserved miR-545 binding site was identified in the TF 3’UTR using TargetScan. (**B**) A dual luciferase reporter assay demonstrated that miR-545 significantly decreased the relative luciferase reporter activity of the pmirGLO-TF-3’UTR. A Western blot assay showed that overexpression of miR-545 significantly decreased TF expression in HT-29 and HCT-116 cells (**C**), but inhibition of miR-545 significantly increased TF expression (**D**). ^*^p < 0.05, ^***^p < 0.001 vs. as indicated.

We identified a conserved miR-545 binding site in the 3’UTR of transferrin (TF) (Figure 4A). A dual luciferase reporter assay demonstrated that miR-545 significantly inhibited the relative luciferase reporter activity of pmirGLO-TF-3’UTR, but no changes were found in the luciferase activity of pmirGLO-TF-3’UTR-Mut (Figure 4B). Furthermore, overexpression of miR-545 decreased, while inhibition of miR-545 increased, TF expression in CRC cells (Figure 4C, 4D). These data indicate that miR-545 targets the TF gene.

### Overexpression of TF abolishes miR-545-induced downregulation of lipid oxidation and iron accumulation

**Figure 5:**
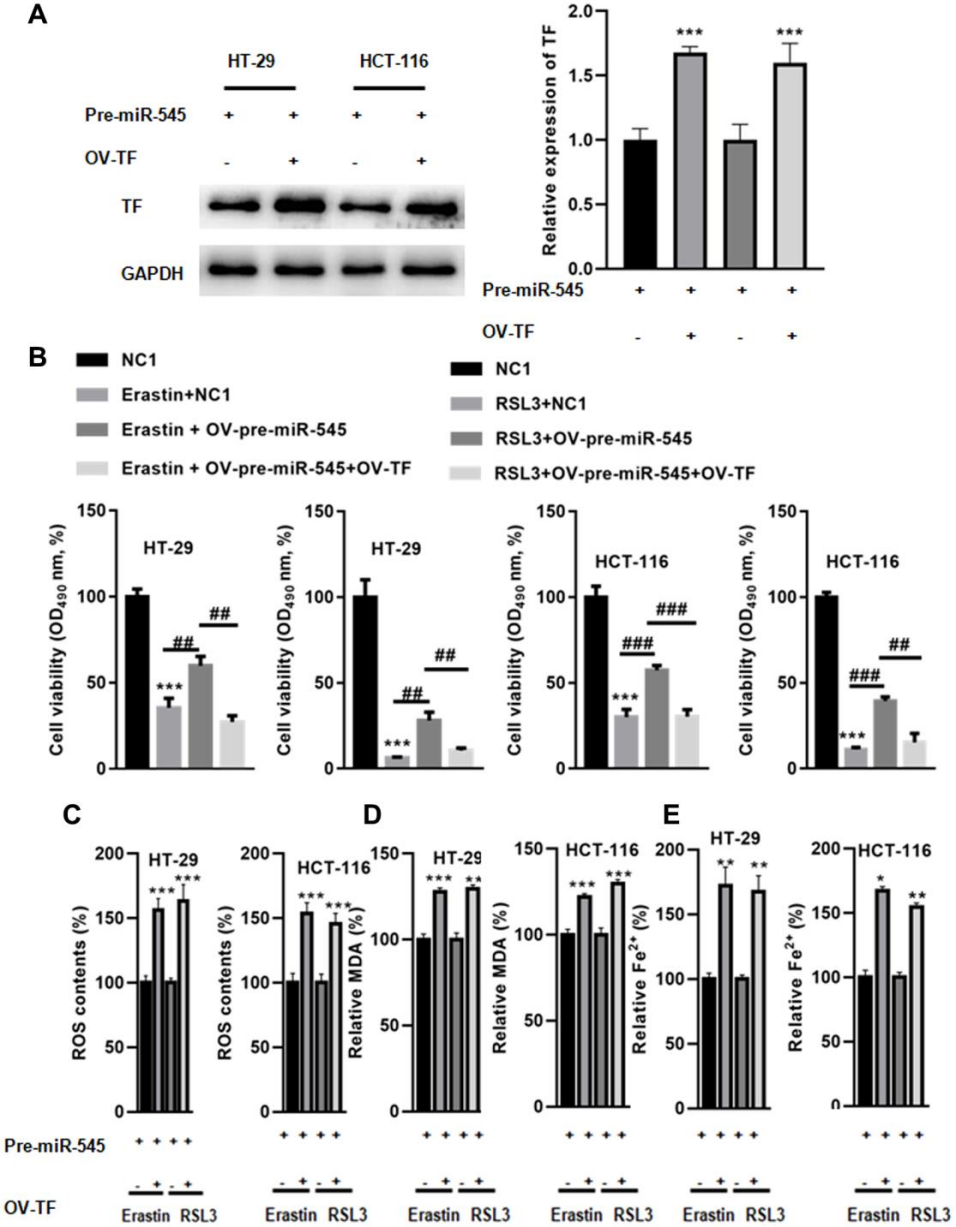
**Overexpression of TF abolished miR-545-induced downregulation of lipid oxidation and iron accumulation.** (**A**) Transfection with OV-TF significantly increased TF expression in HT-29 and HCT-116 cells in the presence of OV-pre-miR-545. (**B**) A CCK-8 assay showed that overexpression of TF further augmented erastin and RSL3-induced reduction of cell survival in HT-29 and HCT-116 cells. (**C**) TF also decreased ROS content in HT-29 and HCT-116 cells treated with erastin and RSL3. (**D**) MDA levels were also reduced in HT-29 and HCT-116 cells transfected with OV-TF. (**E**) Fe^2+^ levels decreased when TF was overexpressed in HT-29 and HCT-116 cells. ^*^p < 0.05, ^**^p < 0.01, ^***^p < 0.001 vs. as indicated.

Next, a rescue experiment was performed to explore whether miR-545 regulated ferroptosis via TF. As shown in Figure 5A, transfection with OV-TF significantly increased TF expression in CR cells in the presence of OV-pre-miR-545. A CCK-8 assay showed that TF overexpression further augmented erastin and RSL3-induced decreases in CRC cell survival (Figure 5B). TF overexpression also decreased ROS content in CRC cells treated with erastin and RSL3 (Figure 5C). Furthermore, MDA levels were reduced in CRC cells transfected with OV-TF (Figure 5D). Similarly, Fe^2+^ levels decreased when TF was overexpressed in CRC cells (Figure 5E). These results indicate that miR-545 inhibits erastin and RSL3-induced CRC cell ferroptosis by inhibiting TF.

### Inhibition of miR-545 decreased tumor growth *in vivo*

**Figure 6:**
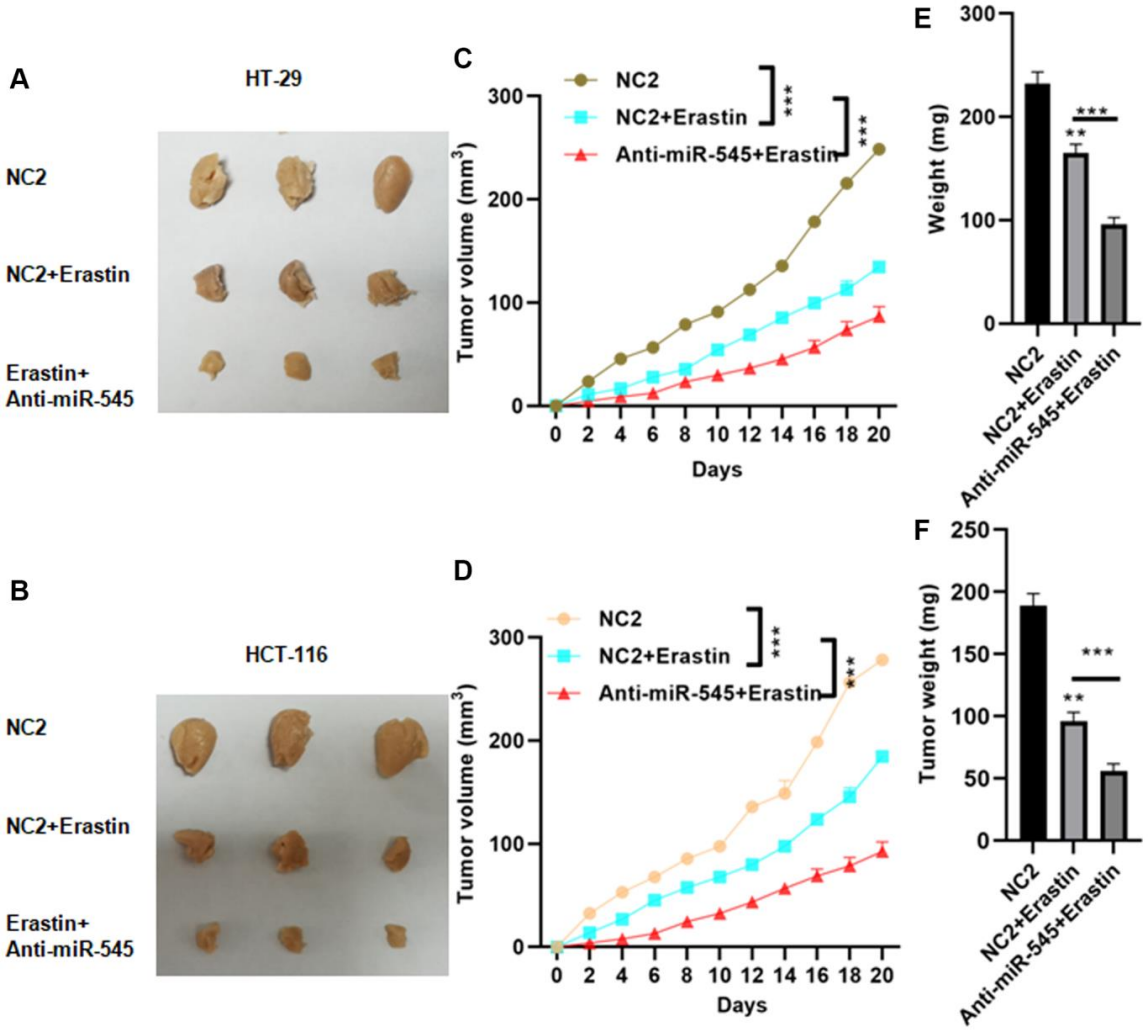
**Inhibition of miR-545 decreased tumor growth *in vivo*.** Inhibition of miR-545 decreased tumor sizes in C57BL/6 mice injected subcutaneously with indicated HT-29 (**A**) or HCT-116 (**B**) cells. Inhibition of miR-545 reduced tumor volumes compared to erastin alone for HT-29 (**C**) and HCT-116 (**D**) cells. Inhibition of miR-545 further reduced tumor weights compared to erastin alone for HT-29 (**E**) and HCT-116 (**F**) cells. ^*^p < 0.05, ^**^p < 0.01, ^***^p < 0.001 vs. as indicated.

Finally, we used lentivirus vectors to achieve a stable and lasting reduction in miR-545 levels in CRC cells. Erastin-treated CRC cells with miR-545 knockdown were then implanted subcutaneously in nude mice. Erastin treatment decreased tumor sizes, and suppression of miR-545 further decreased tumor size (Figure 6A and 6B). The final tumor volume for NC2, NC2+Erastin, and anti-miR-545+Erastin of CRC cells were 248.5 ± 12.3 mm^3^, 134.6 ± 134.6 m^3^, 86.7 ± 9.3 mm^3^ and 267.9 ± 16.1 mm^3^, 184.5 ± 9.3 mm^3^, 92.5 ± 8.7 mm^3^, respectively (Figure 6A and 6B). Similar results were observed for tumor volume and weight (Figure 6C–6F).

## DISCUSSION

Abnormal expression of miRNAs is indicated to participate in tumor development and progression [18, 19]. In this study, we showed that erastin and RSL3 could effectively induce HT-29 and HCT-116 cell death, indicating that CRC cells are susceptible to ferroptosis inducers. Furthermore, our results demonstrated that miR-545 inhibited erastin and RSL3-induced HT-29 and HCT-116 cell death. In contrast, inhibition of miR-545 sensitized HT-29 and HCT-116 cells to canonical ferroptosis induced by erastin and RSL3, which suppress cysteine-dependent GSH synthesis and enhance the production of toxic lipid ROS and lipid oxygen [15]. We observed that both erastin and RSL3 increased lipid oxidation in CRC cells. More importantly, overexpression of miR-545 decreased, while miR-545 inhibition further increased, erastin and RSL3-induced upregulation of ROS and MDA in HT-29 and HCT-116 cells. Interestingly, Fe^2+^ levels were reduced in erastin and RSL3-treated HT-29 and HCT-116 cells, and concomitant inhibition of miR-545 reduced Fe^2+^ levels even further. These findings indicate that miR-545 suppressed ferroptosis in CRC cells, thereby facilitating cancer cell survival.

Ferroptosis is a regulated necrosis via iron-induced accumulation of ROS [20]. In the canonical pathway, inhibition of the peroxidation damage repair system induces ferroptosis [21]. Among the GPX enzymes, only GPX4 may be able to defend biomembranes from peroxidation damage [22]. Non-canonical ferroptosis induction is associated in changes in iron levels. The labile iron pool (LIP) is a small pool that contains iron-related compounds and it induces the production of ROS via Fenton reactions and thereby promote lipid peroxidation [9, 23]. In addition, over activation of heme oxygenase 1 (HMOX1), or increased TF expression may increase the LIP, thereby inducing non-canonical ferroptosis [9].

Interestingly, we identified a conserved miR-545 binding site in the 3′UTR of TF, a crucial iron transport protein. Further study validated that the TF gene was indeed targeted by miR-545. Transferrin receptor 1 binds iron-transferrin complexes at the cell surface, thereby regulating cellular iron uptake [24]. A recent study reported that lapatinib alone or combined use of siramesine increased TF expression, which then increased cell death [25]. Conversely, inhibition of TF reduced cell death and decreased ROS production [25]. These data indicate that increased TF expression can induce ferroptosis.

To determine whether miR-545 suppresses ferroptosis via TF, we overexpressed TF in CRC cells. We found that TF overexpression blocked miR-545-induced increases in ROS, MDA, and Fe^2+^ levels in CRC cells, thereby promoting CRC cell death. These results suggest that miR-545 inhibits ferroptosis in CRC cells by inhibiting TF. An *in vivo* assay indicated that inhibition of miR-545 decreased tumor growth in nude mice treated with erastin, confirming that miR-545 may play an oncogenic role in CRC.

Taken together, our findings illustrate a novel mechanism in which miR-545 inhibits ferroptosis by regulating iron accumulation in CRC cells.

## MATERIALS AND METHODS

### Cell culture

Human normal colon epithelial cell line, NCM460 was purchased from American Type Culture Collection (ATCC, Manassas, VA, USA), and cultured in McCoy's medium (Hyclone; GE Healthcare Life Sciences, Logan, UT, USA). Human CRC cells, including HT-29, HCT-116 and LoVo (ATCC, Manassas, VA, USA), were cultured at 37°C in RPMI-1640 medium (Hyclone; GE Healthcare Life Sciences, Logan, UT, USA) in an incubator with 5% CO_2_. All cell cultures were supplemented with 10% fetal bovine serum (FBS, Gibico), 100 μg/mL penicillin and 100 μg/mL streptomycin (Hyclone; GE Healthcare Life Sciences, Logan, UT, USA).

### Plasmid and lentivirus vectors

Plasmids for miR-545 overexpression (OV-pre-miR-545) and its associated negative control 1 (NC1), as well as miR-545 suppression (anti-miR-545) and corresponding NC2, were constructed by Genchem (Shanghai, China). Vigofect transfection reagent (Vigorous, Beijing, China) was used for the transfection of plasmids into HT-29 and HCT-116 cells according to instructions.

The miR-545 inhibition lentivirus vector (len-anti-miR-545) was constructed by Genchem (Shanghai, China). In brief, HT-29 and HCT-116 cells were seeded in a six-well plate at a density of 10^6^ cells/well. 24 h later, HT-29 cells or HCT-116 cells were transfected with len-anti-miR-545 or NC2 at a titer of 10^9^ pfu/mL for 48 h.

### Cell counting kit-8 (CCK-8) assay

In brief, for CCK-8 assay, HT-29 and HCT-116 cells were inoculated in 96-well plates (10^3^ cells per well) in DMEM culture. After 24 h, 2.5, 5.0, 10.0, 20.0, 40.0 μM erastin or 0.1, 0.5, 1.0, 5.0, 10.0 μM RSL3 was added into for 24 h. Cell viability was examined with the CCK-8 kit (#96992, Sigma-Aldrich; Merck KGaA). In brief, 10 μL CCK8 solution was added into each well at 37^o^C for 3 h and the absorbance was measured at wavelength of 550 nm. All analysis were carried out in three separate experiments.

### RT-PCR

RNAVzol (Vigorous Biotechnology Beijing Co., Ltd., Beijing, China) was used to isolate total RNA from HT-29 or HCT-116 cells. QuantiTect Reverse Transcription Kit (Thermo Fisher Scientific, Inc., Waltham, MA, USA) was used to reverse transcribe RNA into cDNA according to the manufacturer’s instructions. Quantative PCR was performed using SYBR Green Super mix (Bio Rad Laboratories, Inc., Hercules, CA, USA). U6 was used to normalize the samples with the 2^−ΔΔCq^ method [26].

The sequences used in the present study were listed as follows:

miR-545-RT: 5′-GTCGTATCCAGTGCAGGGTCCGAGGTATTCGCACTGGATACGATCATC-3′;

U6-RT: 5′-GTCGTATCCAGTGCAGGGTCCGAGGTATTCGCACTGGATACGACAAAATG-3′;

miR-545-F: 5′-GCTCAGTAAATGTTTATTAG-3′;

U6-F: 5'-GCGCGTCGTGAAGCGTTC-3′;

Universe reverse primer: 5′-GTGCAGGGTCCGAGGT-3′.

### Western blot

Total protein was lysed using a total protein extraction kit (Beijing Solarbio Science and Technology Co., Ltd.) and protein concentration was measured using a BCA protein assay kit (Pierce; Thermo Fisher Scientific, Inc.). 20 μg/lane protein was loaded to the 12% SDS-PAGE and transferred onto polyvinylidene difluoride (PVDF) membranes. Membranes were blocked with 5% non-fat milk for 2 h (Beijing Solarbio Science and Technology Co., Ltd., Beijing, China) at room temperature. Then, the membrane was washed three times with 0.1% TBST (Beijing Solarbio Science & Technology Co., Ltd., Beijing, China) and incubated with primary antibodies at 4°C overnight. After washing with 0.1% TBST for three times, the membranes were incubated with horseradish peroxidase (HRP)-conjugated goat anti-rabbit IgG (Beijing Zhongshan Golden Bridge Biotechnology Co., Beijing, China) for 2 h at room temperature. After washing with 0.1% TBST for three times, enhanced chemiluminescence reagents (Millipore) were used to observe the immunoreactive bands. GAPDH was used as an internal control to normalize the protein. The primary antibodies used in the study were as follows: transferrin (TF) (Abcam, Cambridge, UK), and GAPDH (Cell Signaling Technology, Inc.).

### Dual luciferase reporter assay

To construct pmirGLO-TF-3′UTR or pmirGLO-TF-3’ mutant reporter plasmids, the 3’untranslated region (3′UTR) of TF was cloned into pmirGLO plasmids using *EasyPure*^®^ Genomic DNA Kit or *Fast* Mutagenesis System (TransGen Biotech, Beijing, China). In brief, 293T cells were seeded at 6 well plate at a density of 10^6^ cells/well for 24. After that, the cells were co-transfected with miR-545 mimic/pmirGLO-TF-3′UTR or miR-545 mimic/pmirGLO-TF-3′UTR-Mut using Vigofect transfection reagent (Vigorous, Beijing, China) for 48 h. Dual-Luciferase^®^ Reporter Assay system (Promega Corporation) was used to analyze the relative luciferase activity according to the manufacturer’s protocol.

### Malondialdehyde (MDA) and Fe^2+^ assays

MDA and Fe^2+^ levels were measured using the Lipid Peroxidation MDA Assay Kit (S0131S, Beyotime, Beijing, China) and the iron assay kit (Leagene, Beijing, China), respectively.

### Reactive oxygen species (ROS) quantification

In brief, HT-29 or HCT-116 cells were transfected with NC1/OV-pre-miR-545 or NC2/anti-miR-545 in the presence of erastin or RSL3 for 24 h. After that, the cells were incubated with 2′, 7′-dichlorodihydro-fluorescein diacetate (DCFH-DA) at 37°C for 20 min. After washing with PBS for three times, the cells were re-suspended in 500 μL PBS. Finally, ROS levels were measured using a FACS Calibur flow cytometer equipped with CellQuest software (BD Biosciences).

### Xenograft mouse model

All animal experiments were in accordance with the Guidelines for the Care and Use of Laboratory Animals and were approved by the Animal Research Committee of Zhejiang University School of Medicine.

5-week-old immunodeficient nude mice (male, weight, 16–20 g, *n* = 40 mice for each group) were purchased from Cyagen bio. Co. (Beijing, China). Before experiments, the mice were adapted to the breeding environment for two weeks. All mice were maintained at a 12 h/12 h light/dark cycle with free access to water and food. A total of 5 × 10^6^ HT-29 or HCT-116 cells were suspended in 100 μL PBS and injected subcutaneously into the right posterior flanks of nude mice. After three weeks, the mice were killed and the tumor xenografts were dissected, weighed and fixed in 10% buffered formaldehyde for further IHC analysis. The tumor volumes were calculated using the following formula: a^2^ × b × 0.4 (“a” represents the smallest diameter and “b” represents the diameter perpendicular to “a”).

### Statistical analysis

The values were presented as means ± standard deviation (SD). Independent two-tailed unpaired student’s *t*-tests or one-way ANOVA multiple comparison test were used for comparison of the data. All data were processed using SPSS 20.0 software (Chicago, IL, USA). *P* < 0.05 was considered statistically significant.
